# 术前血浆D-二聚体水平与非小细胞肺癌的相关性分析

**DOI:** 10.3779/j.issn.1009-3419.2019.03.06

**Published:** 2019-03-20

**Authors:** 旭 宋, 丰民 王, 海波 沈, 杰 李, 天军 胡, 振华 杨, 银杰 周, 强 施

**Affiliations:** 315000 宁波，宁波市第二医院 Department of Thoracic Surgery, Ningbo No.2 Hospital, Ningbo 315000, China

**Keywords:** 肺肿瘤, 淋巴结, D-二聚体, Lung neoplasms, Lymph node, D-dimer

## Abstract

**背景与目的:**

随着低剂量螺旋计算机断层扫描（computed tomography, CT）等筛查手段的普及，肺部小结节患者可以早期发现和治疗，但现有的检查手段对于肺小结节良恶性和肺癌淋巴结转移情况的预测均有一定局限性。本研究旨在分析术前血浆D-二聚体水平与非小细胞肺癌（non-small cell lung cancer, NSCLC）患者病理特征的相关性，从而探讨在早期肺癌患者术中进行常规系统性淋巴结清扫的必要性。

**方法:**

回顾性分析567例肺部结节手术患者资料，使用*Kruskal-Wallis*检验、*Spearman*相关系数检测和绘制受试者操作特征曲线（receiver operator characteristic curve, ROC）等统计学方法分析患者术前D-二聚体水平与肺部结节良恶性、肺癌胸膜侵犯、肿瘤最大径、淋巴结转移情况等的相关性。

**结果:**

术前D-二聚体的水平：肺癌患者显著高于肺部良性结节患者（*P* < 0.001）；肺癌伴淋巴结阳性患者显著高于淋巴结阴性患者（*P* < 0.001）；肺癌伴胸膜侵犯阳性患者显著高于胸膜侵犯阴性患者（*P* < 0.001）。肺癌患者的术前D-二聚体水平与淋巴结转移个数呈正相关（*Spearman* Correlation=0.264, *P* < 0.001），与肿瘤最大径呈正相关（*Spearman* Correlation=0.333, *P* < 0.001）。术前血浆D-二聚体水平对T1期NSCLC是否伴有淋巴结转移的最佳诊断指标为112.5 ng/mL。

**结论:**

血浆D-二聚体水平对NSCLC患者的早期诊断、临床分期和预后判断有非常重要的临床意义，并且可以作为术中是否进行淋巴结清扫的参考指标之一。

## Abstract (en)

**Background and objective:**

More and more patients with small pulmonary nodules (SPN) can be found along with the developing of chest low-dose computed tomography (LDCT). With current examinations not all the SPN can be diagnosed to be benign or malignant and not all the malignant nodules can be diagnosed to be lymphatic metastasis. We need to study the correlation between plasma D-dimer count of patients before surgery with pathology features of non-small cell lung cancer (NSCLC).

**Methods:**

The study comprised 567 highly suspected lung cancer patients. Preoperative plasma D-dimer were qualified, and the relationship between plasma D-dimer with pathology features including benign or malignant nodules, tumor size and involvement of lymph nodes was examined using *Kruskal-Wallis* test and *Spearman* correlation coefficients.

**Results:**

The median plasma D-dimer values were statistically higher in NSCLC patients than in those who suffered from benign lung nodules (*P* < 0.001). The median plasma D-dimer values in NSCLC patients with malignant lymph nodes were statistically higher than in those without malignant lymph nodes (*P* < 0.001). An obvious relationship was observed between elevated D-dimer with number of malignant lymph nodes involvement and tumer size. An obvious relationship was observed between elevated D-dimer (> 112.5 ng/mL) and malignant lymph node involvement in stage T1 lung cancer.

**Conclusion:**

The plasma D-dimer maybe useful for early diagnosis, staging and prognosis of the patients with NSCLC. The plasma D-dimer can be one of the indicator to identify what kind of patients need mediastinal lymph node cleaning.

恶性肿瘤和凝血功能障碍存在密切的关系。机体凝血功能的亢进，一方面可导致血栓事件的发生，引起肿瘤患者血栓并发症；另一方面，多种止血、凝血相关因子与肿瘤的发生、发展、转移有着密切的关系^[1]^。有研究^[2-4]^表明，胃肠肿瘤、肺癌、胰腺癌更易引起血液高凝状态。D-二聚体来源于纤溶酶溶解的交联纤维蛋白，是一种特异性的纤溶过程标记物。血浆D-二聚体水平在多种实体肿瘤患者中有显著升高，另有研究^[5]^表明，血浆D-二聚体水平的升高可能提示肺癌患者的不良预后。本文将探讨术前血浆D-二聚体水平与肺癌病理特征的相关性。

## 1 资料与方法

### 1.1 研究对象

回顾性分析宁波市第二医院胸外科2016年1月-2017年8月期间因肺部肿物行手术治疗的567例患者的资料（表 1）。手术方式为：全肺切除、肺叶切除、肺段切除和楔形切除，其中肺癌病例术中均进行了系统性淋巴结清扫。排除标准：（1）术前服用抗凝药物者；（2）肺癌已有远处转移或合并其他恶性肿瘤；（3）术前3个月内曾行手术治疗；（4）细菌或病毒感染活动期；（5）既往曾患血栓性疾病或围手术期发生血栓性疾病；（6）心梗及脑卒中病史。

**1:** 研究对象特征 Characteristics of the patients

Characteristic	Benign nodules	NSCLC without metastasis lymph node	NSCLC with metastasis lymph node
Age (yr)			
Median	57	59	63
Range	25-79	25-85	44-83
Gender			
Male	47	157	40
Female	37	264	22
D-dimer (ng/mL)			
Mean	64.33	111.89	177.5
Range	0-379	0-985	37-579
Metastasis lymph node			
Mean	-	-	3.45
Range	-	-	1-16
Tumor size (cm)			
Mean	-	1.5	3.46
Range	-	0.6-3.5	1.2-11
Pathological type			
Squamous carcinoma	-	39	18
Adenocarcinoma	-	382	44
Pleural invasion			
Positive	-	46	22
Negative	-	375	40
NSCLC: non-small cell lung cancer.			

### 1.2 检测方法

术前抽取受检者清晨空腹静脉血3 mL，经枸橼酸钠抗凝食管混匀，使用自动胶乳增强免疫测定法，测得患者血浆D-二聚体水平，单位为ng/mL。试剂和仪器均为美国贝克曼库尔特实验系统有限公司产品。

### 1.3 统计学方法

实验数据采用SPSS 20.0统计软件处理。经统计分析，D-二聚体水平不符合正态分布，故使用*Kruskal-Wallis*和*Wilcoxon*检验分析肺部良性结节、肺癌淋巴结阴性、肺癌淋巴结阳性三组患者血浆D-二聚体均值的统计学差异，和肺部良性结节、肺癌胸膜侵犯阴性、肺癌胸膜侵犯阳性三组患者血浆D-二聚体均值的统计学差异；分别使用*Spearman*检测分析淋巴结转移数量和肿瘤大小与血浆D-二聚体水平的相关性，通过绘制受试者操作特征曲线（receiver operator characteristic curve, ROC），计算ROC曲线下面积（area under the curve, AUC），得出D-二聚体水平对T1期肺癌伴有淋巴结转移的最佳诊断指标。

## 2 结果

### 2.1 病例特点

如表 1所示，在所有符合研究条件的567例患者中，84例（14.8%）为肺部良性结节，483例（85.2%）为肺癌，其中57例（11.8%）为鳞癌，426例（88.2%）为腺癌。肺癌患者中62例（12.8%）为淋巴结阳性，转移淋巴结个数平均为3.45枚（1枚-16枚），肿瘤最大径平均为3.46 cm（1.2 cm-11 cm），其中22例（35.5%）病理证实为胸膜侵犯。肺癌患者中421例（87.2%）为淋巴结阴性，肿瘤最大径平均为1.5 cm（0.6 cm-3.5 cm），其中46例（10.9%）术后病理证实为胸膜侵犯。

### 2.2 D-二聚体水平在淋巴结阳性及胸膜侵犯患者中显著升高

统计术前D-二聚体水平，肺癌患者（平均为120.31 ng/mL）显著高于肺部良性结节患者（平均为64.33 ng/mL）（*P* < 0.001）；淋巴结阳性患者（平均为177.5 ng/mL）显著高于淋巴结阴性患者（平均为111.89 ng/mL）（图 1A，*P* < 0.001）；胸膜侵犯阳性患者（平均为162.87 ng/mL）显著高于胸膜侵犯阴性患者（平均为113.34 ng/mL）（图 1B，*P* < 0.001）。有研究证明，D-二聚体水平与年龄增长呈正相关，为排除年龄因素的干扰，我们分别统计了年龄≤60岁患者及年龄 > 60岁患者中肺部良性结节、肺癌淋巴结阴性、肺癌淋巴结阳性三组术前D-二聚体水平，发现在年龄≤60岁患者（图 2A，*P* < 0.001）和年龄 > 60岁患者（图 2B，*P* < 0.001）中，三组术前D-二聚体水平均有统计学差异。

**1:**
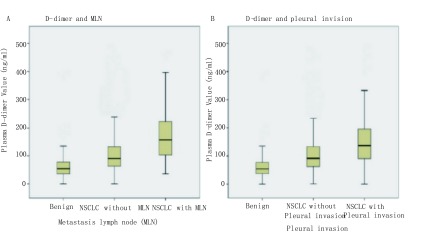
D-二聚体水平与淋巴结转移及胸膜侵犯的相关性。A：使用*Kruskal-Wallis*检验分析肺部良性结节、肺癌淋巴结阴性和肺癌淋巴结阳性三组患者术前血浆D-二聚体水平的均值，使用*Wilcoxon*检验分析肺癌淋巴结阴性和肺癌淋巴结阳性患者术前血浆D-二聚体水平的均值；B：使用*Kruskal-Wallis*检验分析肺部良性结节、肺癌胸膜侵犯阴性、肺癌胸膜侵犯阳性三组患者术前血浆D-二聚体水平的均值，使用*Wilcoxon*检验分析肺癌胸膜侵犯阴性、肺癌胸膜侵犯阳性患者术前血浆D-二聚体水平的均值。 The association between D-dimer and metastasis lymph node, the association between D-dimer and pleural invasion. A: *Kruskal-Wallis* test was used to compare the plasma D-dimer values among the benign group, benign lymph node group and metastasis lymph node group. *Wilcoxon* test was used to compare the plasma D-dimer values between benign lymph node group and metastasis lymph node group; B: *Kruskal-Wallis* test was used to compare the plasma D-dimer values among the benign group, pleural invasion-negative group and pleural invasion-positive group. *Wilcoxon* test was used to compare the plasma D-dimer values between pleural invasion-negative group and pleural invasion-positive group.

**2:**
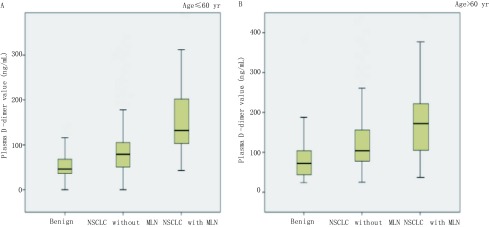
不同年龄组患者中D-二聚体水平变化。使用*Kruskal-Wallis*检验分别分析年龄≤60岁患者（A）、年龄 > 60岁患者（B）中肺部良性结节、肺癌淋巴结阴性和肺癌淋巴结阳性三组患者术前血浆D-二聚体水平的均值。 D-dimer values in patients of different ages. *Kruskal-Wallis* test was used to compare the plasma D-dimer values among the benign group, benign lymph node group and metastasis lymph node group. A: NSCLC patients of 60 years or younger; B: NSCLC patients of more than 60 years.

### 2.3 D-二聚体水平与淋巴结转移个数及肿瘤直径的相关性

肺癌患者的术前D-二聚体水平与淋巴结转移个数呈正相关（图 3A，*Spearman* Correlation=0.264，*P* < 0.001），与肿瘤最大径呈正相关（图 3B，*Spearman* Correlation=0.333，*P* < 0.001）。肿瘤大小、淋巴结转移、胸膜侵犯等情况决定了肺癌的病理分期，所以，术前血浆D-二聚体水平很可能可以预测肺癌病理分期情况。

**3:**
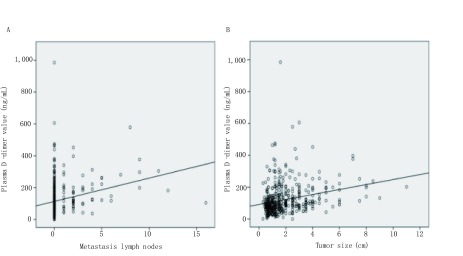
D-二聚体水平与肿瘤病理特征的相关性分析。A：通过*Spearman*检测分析血浆D-二聚体水平与肺癌淋巴结转移个数之间的关系；B：通过*Spearman*检测分析血浆D-二聚体水平与肺癌肿瘤最大径之间的关系。 The association between D-dimer and pathological features of tumor. A: *Spearman* correlation was used to assess the association between D-dimer values and number of metastasis lymph nodes; B: *Spearman* correlation was used to assess the association between D-dimer values and tumor size.

### 2.4 D-二聚体水平对淋巴结转移的预测

在术中快速病理切片证实为T1期肺癌的患者中，血浆D-二聚体水平可以作为预测是否伴有淋巴结转移的重要指标。我们已经证实，肺癌伴淋巴结转移的患者术前血浆D-二聚体平均水平显著高于肺癌不伴淋巴结转移的患者（图 1A），而且术前血浆D-二聚体水平与淋巴结转移个数呈正相关（图 3A）。所以，D-二聚体水平可能可以预测临床诊断为早期肺癌的患者是否伴有淋巴结转移。我们针对术中快速病理切片证实为T1期的肺癌患者（363例）中，淋巴结阴性者（241例）和淋巴结阳性者（22例）的术前D-二聚体水平，绘制ROC，通过计算AUC，得出D-二聚体对肺癌是否伴有淋巴结转移的最佳诊断指标（图 4，AUC=0.76，95%CI：0.648-0.872，*P* < 0.001）：当临界值为112.5 ng/mL时，淋巴结转移的阳性预测比例为0.77，阴性预测比例为0.73。所以，D-二聚体水平对于肺癌患者是否伴有淋巴结转移的预测有较高的特异性和敏感性。

**4:**
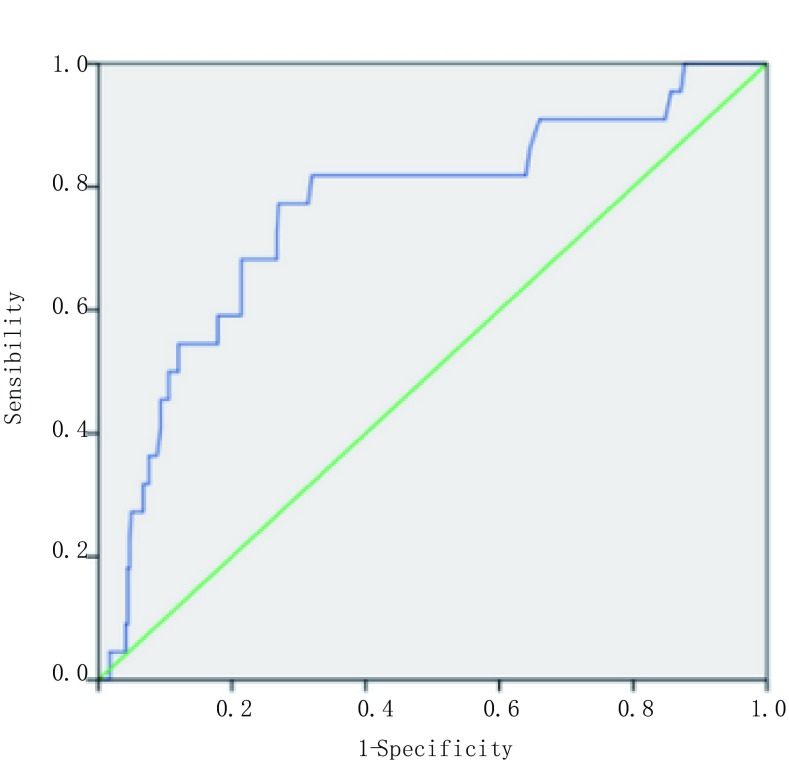
D-二聚体水平对淋巴结转移的预测。针对肺癌淋巴结阴性患者和肺癌淋巴结阳性患者的术前D-二聚体水平，绘制受试者操作特征曲线（ROC），通过计算ROC曲线下面积（AUC），得出D-二聚体水平对肺癌是否伴有淋巴结转移的最佳诊断指标（AUC=0.76, 95%CI: 0.648-0.872, *P* < 0.001）。 D-dimer could be used to predict metastasis lymph node. Area under the receiver operating characteristic curve value was used to predict NSCLC patients with or without metastasis lymph node (AUC=0.76, 95%CI: 0.648-0.872, *P* < 0.001).

## 3 讨论

肺癌患者有凝血机制的异常，血液处于高凝状态，有以下几点原因：①肺癌组织本身能分泌一些促凝血物质，如促血小板聚集物质，使机体处于高凝状态，具有高血栓形成倾向；②被癌细胞浸润的血管内膜丧失了抗血栓形成的能力，使得血栓容易形成；③反应性白细胞释放的物质的刺激作用；④血液循环中癌细胞的直接作用^[6]^。D-二聚体是体内高凝状态和纤溶亢进分子标志物之一，是直接反映止血酶和纤溶酶生成的指标。有多项研究^[5, 7, 8]^证明，D-二聚体与肺癌的发生、发展关系密切。但这些既往的研究多集中在D-二聚体肺癌术后生存率或化疗、放疗后生存率的相关性上，针对D-二聚体与肺部结节病理特征相关性的研究相对较少^[9]^。

在本研究中，我们针对肺癌的各项病理特征与术前D-二聚体水平的相关性做了更深入的分析，试图探讨术前D-二聚体水平对肺部结节患者术前病情评估的意义，以指导肺部结节患者治疗方案的选择。我们的研究结果表明，肺癌患者的D-二聚体水平显著高于肺部良性结节患者，且随着病理证实的阳性淋巴结数量的增多和肿瘤体积的增大，患者术前D-二聚体水平亦随之增多，这提示血浆D-二聚体水平可能可以在术前预测肺癌的病理分期和预后情况，并可作为肺部结节术前诊断、肺癌病理分期、预后判断的重要预测指标。这可能是因为，处于增殖状态的肿瘤细胞，其局部的浸润、转移状态，加重了其对纤维蛋白溶解系统和血液凝固系统的影响^[10]^。

随着低剂量CT等筛查手段的普及，越来越多的肺部结节得以检出，由于胸腔镜手术技术的日益成熟，很多肺癌患者也可早期发现和早期手术治疗。目前对于肺部结节良恶性的判断主要依靠影像学检查结果，其敏感性和特异性均有一定的局限性，肺部结节的确诊主要依靠术中快速冰冻病理诊断，临床工作中尚需要更多的参考指标来协助术前的诊断。肺癌的分期及淋巴结转移情况主要根据术中系统性淋巴结清扫的结果。但临床实际工作中早期肺癌患者手术淋巴结阳性检出率较低，多数患者无法从系统性淋巴结清扫中获益，而且过多清扫淋巴结，尤其是纵隔淋巴结，可增加术中出血、术后引流量，影响支气管血供，并加重患者术后咳嗽等症状，延长住院时间。我们的研究结果表明术前D-二聚体水平与肺癌的淋巴结转移、胸膜侵犯、肿瘤最大径等病理特征密切相关，故结合术前血浆D-二聚体水平和肿瘤标志物、术前影像学检查等结果，可以决定术中是否需要系统性的纵隔淋巴结清扫。我们的研究证实，当术前D-二聚体水平的临界值为112.5 ng/mL时，淋巴结转移阳性预测有较高的特异性（0.73）和敏感性（0.77）。故结合其他临床检查指标，可对肺癌淋巴结转移情况获得更精确的预测结果。

总之，血浆D-二聚体水平对肺癌患者的早期诊断、临床分期和预后判断有非常重要的临床意义。可作为手术方式选择和预后判断的重要参考指标。
